# Is admission lactate an independent prognostic marker or a reflection of injury severity in severe traumatic brain injury?

**DOI:** 10.3389/fneur.2026.1920098

**Published:** 2026-10-19

**Authors:** Fuli Yan, Hao Liu, Mingang Liang, Wenya Gao, Lun Dong, Jian Wu, Jun Zhang, Mingfei Yang

**Affiliations:** 1 Graduate School, Qinghai University, Qinghai, China; 2 Department of Neurosurgery, Clinical Medical College of Yangzhou University, Jiangsu, China; 3 Department of Neurosurgery, Huai'an Clinical Medical College of Jiangsu University, Jiangsu, China; 4 Huai'an Hospital of Huai'an City, Jiangsu, China; 5 Department of Neurosurgery, Xinhua Hospital Affiliated to Shanghai Jiao Tong University School of Medicine, Shanghai, China; 6 Department of Neurosurgery, Qinghai Provincial People's Hospital, Qinghai, China; 7 National Regional Medical Center for Qinghai (Qinghai Hospital, The First Affiliated Hospital of Xi'an Jiaotong University), Xining, China

**Keywords:** 30-day mortality, prognostic marker, rotterdam CT score, severe traumatic brain injury, venous blood lactate

## Abstract

**Objective:**

Although admission lactate is associated with poor outcomes after severe traumatic brain injury, its independent prognostic value remains uncertain. This study aimed to determine whether admission lactate is an independent prognostic marker or primarily a reflection of injury severity.

**Methods:**

In a two-center retrospective study of patients with sTBI (GCS ≤ 8) (development cohort, *n* = 287; external validation cohort, *n* = 185), we assessed whether the association between admission lactate and 30-day mortality remained independent of established severity markers (age, GCS, pupillary reactivity, and Rotterdam CT score) and quantified the incremental prognostic value of lactate beyond this severity baseline, with external validation. Exploratory *in vitro* experiments in hypoxic SH-SY5Y cells were performed as a secondary analysis to examine cellular responses to lactate under hypoxic conditions.

**Results:**

Thirty-day mortality was 42.2% in the development cohort and 36.8% in the external validation cohort. Admission lactate was significantly associated with mortality on univariable analysis (OR per mmol/L, 1.50; 95% CI, 1.30–1.73). After adjustment for severity markers, the adjusted OR was 1.19 per mmol/L (95% CI, 1.00–1.42; *P* = 0.051), indicating a positive association with limited precision. Consistent with this finding, lactate provided minimal incremental prognostic value beyond the severity baseline (ΔAUC, +0.005; *P* = 0.31), and these results were reproduced in the external validation cohort. Nevertheless, *in vitro* experiments suggested a potential biological activity of lactate that warrants further investigation.

**Conclusion:**

In these two selected adult sTBI cohorts, admission lactate was associated with 30-day mortality but provided little incremental prognostic information beyond established neurological severity markers. Its role should therefore be interpreted as adjunctive rather than as a stand-alone prognostic factor. The exploratory *in vitro* findings warrant further mechanistic investigation.

## Graphical Abstract

**Figure:**
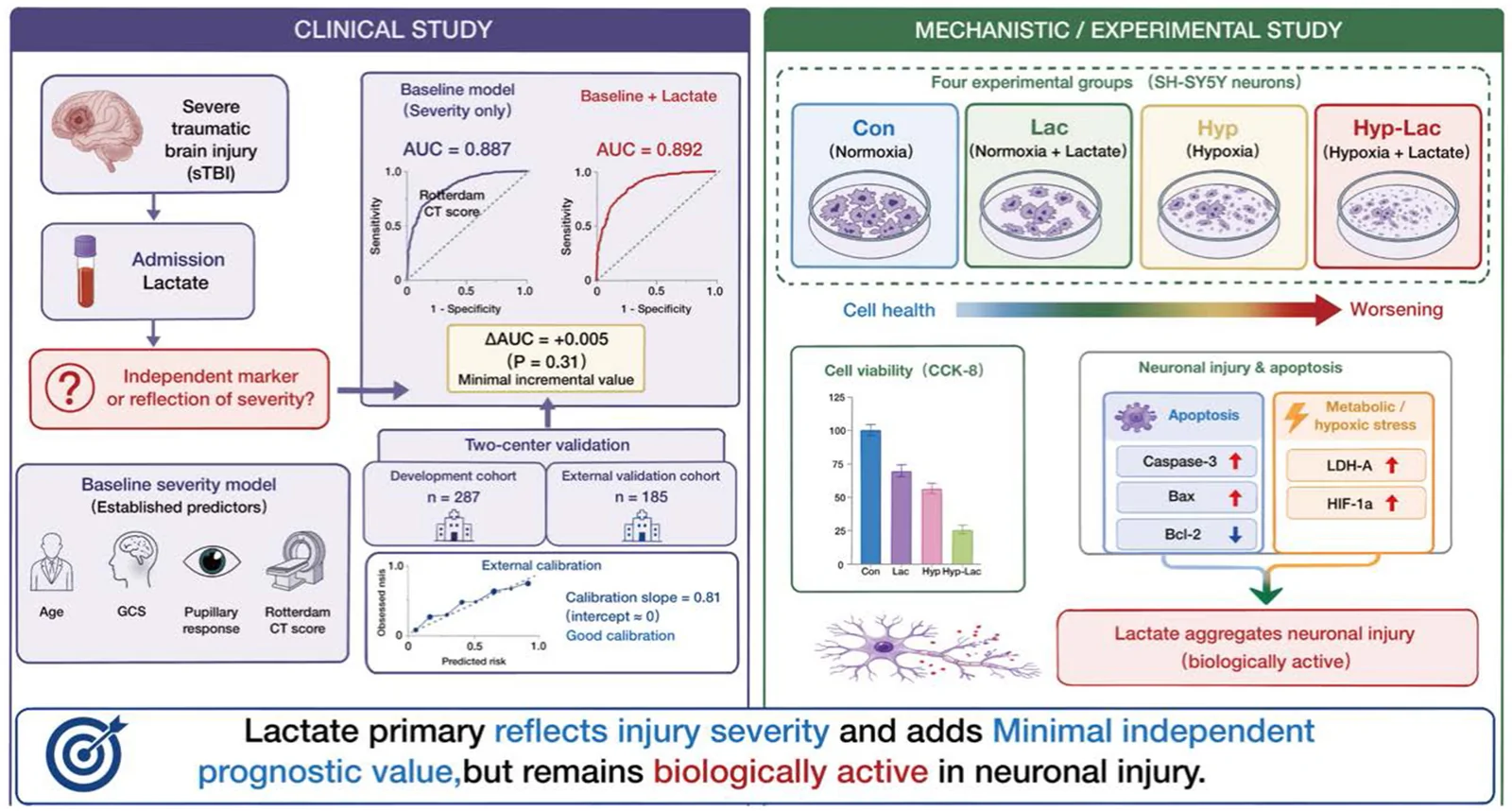
This study examined whether admission lactate is an independent prognostic marker in severe traumatic brain injury (sTBI) or primarily reflects injury severity. In a two-center study (development cohort, n = 287; external validation cohort, n = 185), admission lactate showed little incremental prognostic value beyond age, GCS, pupillary reactivity, and Rotterdam CT score, with some attenuation of calibration on external validation. sTBI, severe traumatic brain injury; GCS, Glasgow Coma Scale; CT, computed tomography; AUC, area under the curve; ΔAUC, change in area under the curve.

## Introduction

Severe traumatic brain injury (sTBI) remains a leading cause of mortality and long-term disability worldwide, and constitutes a particularly heavy clinical and socioeconomic burden in China (1, 2). A defining feature of sTBI is that death is concentrated within the first days after injury, when the primary mechanical insult is compounded by an evolving cascade of secondary injury—mitochondrial dysfunction, excitotoxicity, neuroinflammation, and oxidative stress—that progresses during intensive care (3–5). Because this early phase is both the most lethal and the most amenable to intervention, accurate identification of patients at highest risk of early death is central to triage, monitoring intensity, and resource allocation. Reliable risk stratification at the time of admission, however, remains clinically challenging.

Established prognostication relies on a compact set of severity markers—age, the Glasgow Coma Scale (GCS), pupillary reactivity, and computed tomography (CT) findings—which together underpin guideline-based management and validated outcome models (6–8). Although these markers characterize neurological and structural injury well, they describe the anatomical and neurological consequences of trauma rather than the acute metabolic disturbance that accompanies it; moreover, pupillary examination and CT grading are not always immediately obtainable or unambiguous in the hyperacute setting. These limitations have motivated the search for rapidly measurable circulating markers capable of reflecting the systemic physiological derangement that follows severe injury (9).

Among candidate circulating markers, serum lactate is particularly attractive, being inexpensive, near-universally available, and obtainable within minutes as a product of anaerobic metabolism and tissue hypoperfusion (10, 11). Its prognostic appeal is supported by a large adult multi-center cohort, in which admission lactate emerged as an independent risk factor for 1-week mortality in sTBI (12). Yet the strength of this independence is uncertain. This concern is amplified by studies that do not adjust at all: a large single-center series identified lactate as a “strong predictor” of poor outcome using univariable analyses alone, while explicitly cautioning that lactate cannot be interpreted in isolation given the many confounders that simultaneously elevate lactate and mortality (13). The most direct test of this question comes from a recent severity-balanced TBI cohort, in which admission lactate—alongside lactate clearance and variability—lost its predictive value after adjustment, and only longitudinal, time-weighted lactate remained independently associated with mortality (14). Taken together, these findings expose a more fundamental uncertainty: the prognostic standing of admission lactate in sTBI remains undefined—it is unclear whether lactate constitutes a genuinely independent prognostic marker, or whether it is largely a biochemical reflection of injury severity that adds little once severity is properly accounted for. This question has remained open in part because prior studies seldom adjusted for the principal determinants of neurological injury severity—particularly pupillary reactivity and CT grading—rarely performed independent external validation, and almost never quantified the incremental value of lactate beyond an established severity baseline. Clarifying this standing is clinically consequential: it determines whether lactate deserves a place as an independent prognostic factor, or should instead be regarded as one readily available signal among the broader markers of severity.

To address this gap, we conducted a two-center retrospective study with independent external validation, in which the lactate–mortality association was progressively adjusted for established severity markers and the incremental discriminative value of lactate over this severity baseline was quantified. Accordingly, the primary objective of this study was to determine the incremental prognostic value of admission lactate beyond established neurological severity markers, with independent external validation. Separately, as an exploratory secondary objective, we used a hypoxic SH-SY5Y cell model to characterize cellular responses to lactate under hypoxic conditions.

## Methods

### Study design and participants

This was a two-center retrospective cohort study conducted at the Department of Neurosurgery of the Clinical Medical College of Yangzhou University and the Department of Neurosurgery of Qinghai Provincial People's Hospital. Patients treated at Yangzhou University between January 2018 and December 2022 constituted the development cohort (*n* = 287), and patients treated at Qinghai Provincial People's Hospital between January 2019 and December 2023 constituted the independent external validation cohort (*n* = 185). All patients were consecutively enrolled during the respective study periods if they met the eligibility criteria. The study protocols were approved by the Institutional Review Board of the Clinical Medical College of Yangzhou University (Approval No. 2020KY-147) and the Ethics Committee of Qinghai Provincial People's Hospital (Approval No. 2023-003); the requirement for informed consent was waived owing to the retrospective design. The study was conducted in accordance with the Declaration of Helsinki and is reported following the TRIPOD statement for prediction-model studies (15). Patients from one center constituted the development cohort and those from the other the independent external validation cohort.

Inclusion criteria were: (1) a clear history of trauma with a clinical diagnosis of sTBI (GCS score ≤ 8); (2) age >18 years; (3) time from injury to admission <12 h; (4) admission to the neuro-intensive care unit (NICU); and (5) serum lactate measured immediately on admission. Exclusion criteria were: (1) cardiac arrest requiring resuscitation; (2) multiple systemic injuries; (3) severe infection, shock, severe hepatic or renal insufficiency, or malignancy; (4) repeated blood transfusion; (5) craniotomy performed >5 h after injury; and (6) coexisting hypertension and/or diabetes mellitus.

These exclusion criteria were prespecified to reduce major extracranial, hemodynamic, and metabolic influences on admission lactate and to improve the homogeneity of the study population. Cardiac arrest, multiple systemic injuries, shock, severe infection, major hepatic or renal dysfunction, and repeated transfusion may substantially alter lactate production or clearance independent of neurological injury. Hypertension and/or diabetes were excluded according to the original study protocol to reduce baseline metabolic heterogeneity. The craniotomy-timing criterion was intended to restrict the cohort to patients managed within a relatively comparable early treatment window.

A flow diagram detailing the number of patients excluded for each criterion at each center is provided in Supplementary Figure S1. Briefly, in the development cohort, 412 patients with sTBI were initially screened; 125 were excluded for the following reasons: cardiac arrest (*n* = 8), multiple systemic injuries (*n* = 34), severe infection/shock/organ insufficiency/malignancy (*n* = 19), repeated blood transfusion (*n* = 12), craniotomy >5 h after injury (*n* = 27), and coexisting hypertension/diabetes (*n* = 25). In the external validation cohort, 268 patients were screened; 83 were excluded by the same criteria: cardiac arrest (*n* = 5), multiple systemic injuries (n=20), severe infection/shock/organ insufficiency/malignancy (*n* = 15), repeated blood transfusion (*n* = 10), craniotomy >5 h after injury (*n* = 18), and coexisting hypertension/diabetes (*n* = 15). The development and external validation cohorts comprised 287 and 185 patients, respectively.

### Study settings

This study was conducted at two neurosurgical centers in China with distinct geographic and healthcare settings. The development cohort was treated at the Department of Neurosurgery, Clinical Medical College of Yangzhou University, a tertiary referral center serving a predominantly urban population in the Yangtze River Delta region. Patients are typically transported directly to the hospital by emergency medical services, with prehospital transport times generally <60 min. Neurocritical care is provided in a general intensive care unit with neurosurgical consultation.

The external validation cohort was treated at the Department of Neurosurgery, Qinghai Provincial People's Hospital, a tertiary referral center serving a large catchment area including both urban and rural populations across the Qinghai-Tibet Plateau. Prehospital transport times are often prolonged due to the vast geographic area and mountainous terrain. This center has a dedicated neuro-intensive care unit with standardized intracranial pressure monitoring protocols and a specialized neurocritical care team. Key differences between the two centers are summarized in Supplementary Table S5.

### Data collection and variables

A trained clinical researcher collected admission data using a standardized data-collection form. To prevent outcome leakage, only variables available at the time of admission were used as predictors: age, sex, body weight, time from injury to admission, admission body temperature, admission GCS, pupillary reactivity (graded as both reactive, one fixed, or both fixed), the Rotterdam CT score derived from the admission head CT, emergency craniotomy, and admission venous serum lactate. Serum lactate was analyzed as a continuous variable (per 1 mmol/L).

### Lactate measurement

Admission lactate was defined as the first venous blood lactate measurement obtained immediately upon arrival in the emergency department, before in-hospital intravenous fluid resuscitation or blood transfusion and prior to surgical intervention. Samples were processed in the central laboratory of each center according to routine clinical protocols. Of note, both centers used the same blood gas analyzer model (RAPIDLab 1200, Siemens Healthineers) with an enzymatic method for lactate quantification, ensuring comparability of lactate measurements between the two cohorts.

### Outcomes

The primary outcome was all-cause mortality within 30 days of admission. Thirty-day vital status was ascertained from hospital medical records and post-discharge follow-up information, including readmission records, death registry records, and telephone confirmation when required. Thirty-day vital status was available for all patients in both cohorts. Secondary outcomes were in-hospital mortality and functional outcome at hospital discharge, assessed by the modified Rankin Scale (mRS); patients who died in hospital were assigned an mRS of 6. Withdrawal of life-sustaining treatment (WLST), together with its timing and documented reason, was recorded to enable a prespecified sensitivity analysis.

### Missing data

Missing values in admission covariates were addressed using multiple imputation by chained equations, performed separately within the development and external validation cohorts to preserve the independence of the external validation dataset. Twenty imputed datasets were generated for each cohort. The imputation model included age, sex, body weight, injury-to-admission time, body temperature, admission GCS, pupillary reactivity, Rotterdam CT score, emergency surgery, admission lactate, and 30-day mortality. The outcome was included in the imputation model but was not itself imputed. Regression estimates were pooled across imputations using Rubin's rules. Model-performance measures were calculated within each of the 20 imputed datasets and subsequently summarized across imputations. Complete-case analysis was performed as a sensitivity analysis. The extent and distribution of missing data according to cohort and 30-day outcome are summarized in Supplementary Table S4.

### Statistical analysis: clinical cohorts

Normality of continuous variables was assessed with the Shapiro–Wilk test. Continuous variables are presented as mean ± standard deviation (compared by independent-samples *t*-test) or median (interquartile range; Mann–Whitney U test), and categorical variables as counts (percentages; χ^2^ test). The lactate–mortality association was first assessed by univariable logistic regression and then by multivariable logistic regression adjusting for a prespecified severity baseline comprising age, admission GCS, pupillary reactivity, and Rotterdam CT score; odds ratios (OR) are reported per 1 mmol/L increase with 95% confidence intervals (CI).

The adjustment set was prespecified based on established prognostic models in sTBI. This set represents the core neurological severity indicators used in routine clinical prognostication. We chose this focused adjustment strategy to directly address whether lactate adds incremental information beyond established severity markers. Additional circulatory and metabolic variables were not included in the primary model because they were incompletely available in both cohorts. Therefore, the present analysis was intended to evaluate the incremental prognostic value of lactate beyond a selected neurological severity model rather than to establish its causal independence from systemic physiological disturbances. GCS was assessed using the total score before sedation or paralytic administration whenever possible; if administered, the pre-intubation GCS or the motor component was used as per standard clinical practice.

Three models were specified *a priori*: lactate alone, the severity baseline, and the baseline plus lactate. Discrimination was quantified by the area under the receiver operating characteristic curve (AUC), and nested models were compared using the DeLong test. The incremental value of lactate over the severity baseline was assessed in the development cohort by the change in AUC (ΔAUC), the continuous net reclassification improvement (NRI), and the integrated discrimination improvement (IDI). Overall prediction error was assessed using the Brier score, and 95% confidence intervals were reported for discrimination, incremental-value, and calibration estimates where applicable. Model optimism was corrected by bootstrap internal validation (500 resamples), yielding an optimism-corrected C-index and calibration slope. Clinical utility was evaluated using decision-curve analysis. The displayed threshold-probability range of 0–0.60 was selected to encompass clinically plausible risk thresholds around and above the observed 30-day mortality rates in both cohorts, while avoiding emphasis on the less informative extreme upper-risk range. The relationship between admission lactate and 30-day mortality was modeled using restricted cubic splines with four knots placed at the 5th, 35th, 65th, and 95th percentiles of the lactate distribution (0.78, 2.98, 4.40, and 7.49 mmol/L, respectively), using the cohort median (3.7 mmol/L) as the reference value. Nonlinearity was formally assessed by comparing the spline model with the corresponding linear model. The linearity assumptions for age and admission GCS were assessed similarly using restricted cubic splines. The final multivariable logistic regression model for predicting 30-day mortality included age, admission GCS, pupillary reactivity (categorical: 0 = both reactive, 1 = one fixed, 2 = both fixed), Rotterdam CT score (continuous, 2–6), and admission lactate. The model coefficients and intercept were: Intercept: β*0*= −3.861, Age: β1 = 0.018, Admission GCS: β_2_ = −0.223, Pupillary reactivity: β3 = 0.777, Rotterdam CT score: β4 = 0.914, and Admission lactate: β5 = 0.173. The predicted probability of 30-day mortality was calculated as: *P* = 1 / [1 + exp–(−3.861 + 0.018 × Age – 0.223 × GCS + 0.777 × Pupil + 0.914 × Rotterdam + 0.173 × Lactate)]. For example, for a 60-year-old patient with an admission GCS of 6, one fixed pupil (Pupil = 1), a Rotterdam CT score of 4, and an admission lactate level of 4.0 mmol/L, the model yields an estimated 30-day mortality probability of approximately 73.0%. The baseline-plus-lactate model developed in the development cohort was then applied, with its original coefficients, to the external validation cohort, where discrimination and calibration were assessed. Two prespecified sensitivity analyses were performed: exclusion of WLST patients and complete-case analysis. Analyses were performed in R version 4.3 and GraphPad Prism 9.1; a two-sided *P* < 0.05 was considered statistically significant.

### *In vitro* experiments

To explore the biological activity of lactate under hypoxic conditions, we performed preliminary *in vitro* experiments using undifferentiated human SH-SY5Y neuroblastoma cells (ATCC CRL-2266). Cells were maintained in DMEM/F12 supplemented with 10% fetal bovine serum and 1% penicillin–streptomycin at 37 °C in a humidified atmosphere containing 5% CO_2_. Based on CCK-8 viability assays across graded sodium lactate concentrations, 5 mmol/L was selected for subsequent experiments as it approximated the admission lactate concentrations observed in non-survivors. Hypoxia was induced at 1% O_2_ for 12 h. Cells were assigned to four groups: normoxic control, lactate alone (5 mmol/L), hypoxia alone, and hypoxia plus lactate. Apoptosis was assessed by flow cytometry, and protein expression of Bax, Bcl-2, Caspase-3, HIF-1α, and LDH-A was examined by Western blot. All experiments were performed in three independent replicates. For the four-group experiments, data were analyzed using two-way ANOVA with hypoxia and lactate as fixed factors, followed by Tukey's multiple-comparison test where appropriate. Concentration-response experiments were analyzed using one-way ANOVA followed by Tukey's multiple-comparison test. These experiments were designed as exploratory observations to generate hypotheses, not to establish definitive mechanistic conclusions.

## Results

### Cohort characteristics and admission lactate

The development and external validation cohorts comprised 287 and 185 patients with sTBI, respectively (Table 1). Thirty-day mortality was 42.2% (121/287) in the development cohort and 36.8% (68/185) in the external validation cohort. In-hospital mortality was 39.7% (114/287) and 35.1% (65/185), respectively, and the median discharge mRS was 4.0 (IQR, 2.0–6.0) in both cohorts. In both cohorts, non-survivors were older and more severely injured than survivors, with lower admission GCS, more frequently impaired pupillary reactivity, and higher Rotterdam CT scores (Supplementary Tables S1–S2). Consistent with these severity differences, admission serum lactate was significantly higher in non-survivors than in survivors, and this difference was reproduced across both centers: 4.5 (3.4–5.6) vs. 3.0 (1.8–4.3) mmol/L in the development cohort (Figure 1A) and 4.2 (3.4–5.8) vs. 2.6 (1.6–3.8) mmol/L in the external validation cohort (Figure 1B) (both *P* < 0.001). At the univariable level, therefore, higher admission lactate was clearly associated with 30-day mortality.

**Table 1:** Baseline characteristics of the development and external validation cohorts.

Variables	Development cohort (*n* = 287)	External validation cohort (*n* = 185)
Age, years (mean ± SD)	52.2 ± 15.1	54.0 ± 15.5
Male sex, *n* (%)	233 (81.2)	133 (71.9)
Weight, kg (mean ± SD)	66.4 ± 12.6	64.1 ± 11.5
Injury-to-NICU time, h, median (IQR)	3.0 (2.0–4.5)	3.5 (2.5–5.5)
Temperature, °C (mean ± SD)	36.7 ± 0.6	36.7 ± 0.6
Admission GCS, median (IQR)	6.0 (5.0–7.0)	7.0 (5.0–8.0)
Pupillary reactivity (both/one/none reactive), *n*	153/85/23	96/33/24
Rotterdam CT score, median (IQR)	3.0 (2.0–4.0)	3.0 (2.0–3.0)
Emergency surgery, *n* (%)	175 (61.0)	113 (61.1)
Admission lactate, mmol/L, median (IQR)	3.7 (2.3–5.0)	3.2 (2.1–4.6)
30-day mortality, *n* (%)	121 (42.2)	68 (36.8)

**Figure 1:**
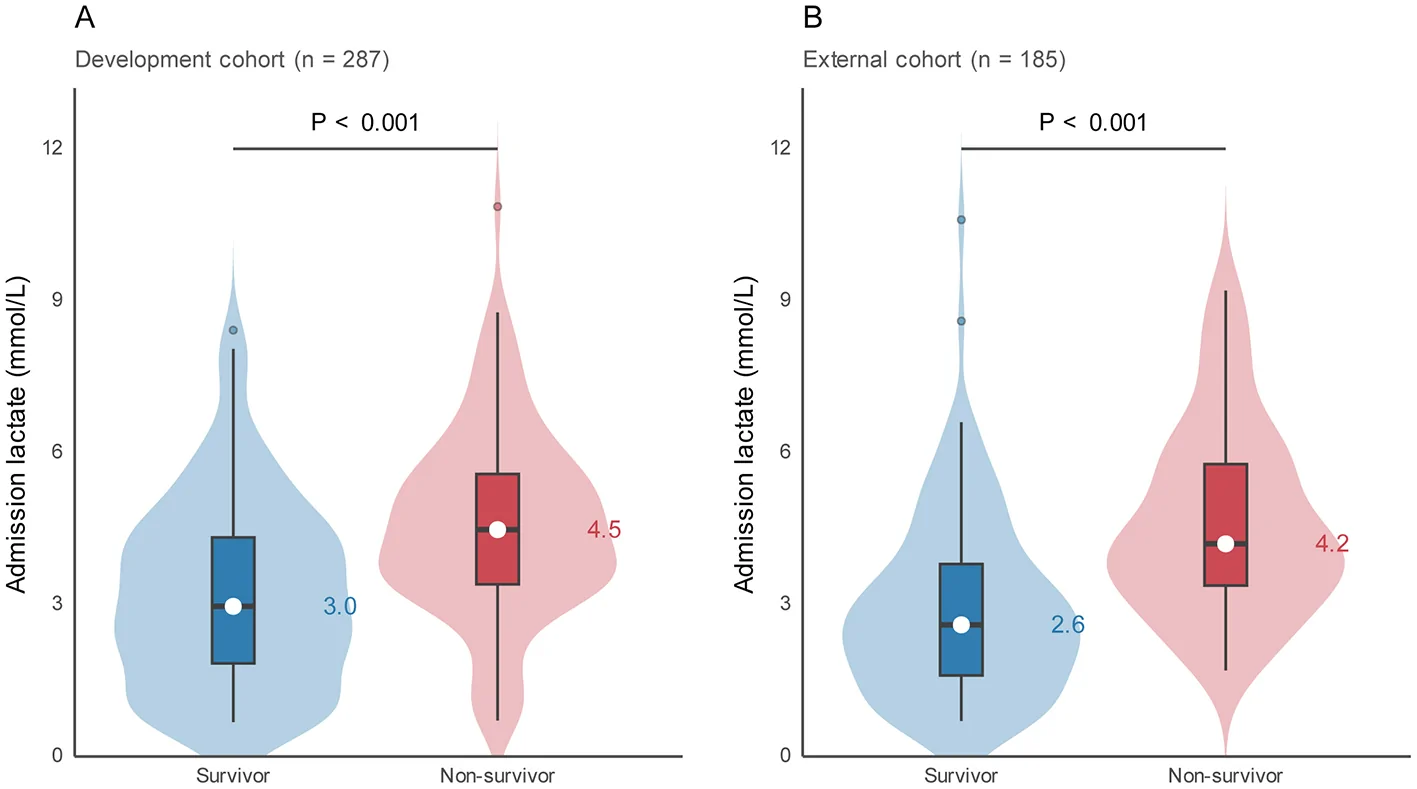
Association between admission serum lactate and 30-day mortality in patients with severe traumatic brain injury. Violin plots showing the distribution of admission serum lactate in survivors and non-survivors in the development cohort **(A)** and the external validation cohort **(B)**. In both cohorts, non-survivors had significantly higher admission lactate than survivors. The central white dot and box indicate the median and interquartile range (IQR). Statistical significance was assessed using the Mann–Whitney U test.

WLST occurred in 40 patients (13.9%) in the development cohort and 27 patients (14.6%) in the external validation cohort. When stratified by 30-day outcome, WLST was more frequent among non-survivors than survivors in both cohorts (30/121 [24.8%] vs. 10/166 [6.0%] in the development cohort and 24/68 [35.3%] vs. 3/117 [2.6%] in the external validation cohort). The timing and documented reasons for WLST according to center and 30-day outcome are summarized in Supplementary Table S6. In the prespecified sensitivity analysis excluding patients who underwent WLST, the association between admission lactate and 30-day mortality remained directionally consistent and was numerically stronger (adjusted OR, 1.23; 95% CI, 1.01–1.50; *P* = 0.036; Supplementary Table S3).

### Lactate is associated with mortality but adds little once injury severity is accounted for

On univariable logistic regression, admission lactate was associated with 30-day mortality (OR per 1 mmol/L, 1.50; 95% CI, 1.30–1.73; *P* < 0.001). After adjustment for the prespecified severity baseline of age, admission GCS, pupillary reactivity, and Rotterdam CT score, the adjusted OR was 1.19 per mmol/L (95% CI, 1.00–1.42; *P* = 0.051). In the complete-case sensitivity analysis, the association was directionally consistent (adjusted OR, 1.27; 95% CI, 1.04–1.56; *P* = 0.019). This estimate suggests a positive association whose precision is limited by the confidence interval just crossing unity, rather than evidence of no effect. Within the adjusted model, pupillary reactivity (OR, 2.18; 95% CI, 1.23–3.85) and Rotterdam CT score (OR, 2.50; 95% CI, 1.85–3.36) were the dominant predictors, whereas GCS was no longer independently significant—consistent with the overlapping severity information shared among these neurological and imaging markers (Table 2). This pattern was mirrored in discrimination (Figure 2). Used alone, lactate discriminated 30-day mortality only moderately in the development cohort (AUC, 0.711; 95% CI, 0.650–0.768), well below the severity baseline (AUC, 0.887; 95% CI, 0.845–0.929). Adding lactate to the baseline changed discrimination only marginally (AUC, 0.892; 95% CI, 0.862–0.922; ΔAUC, +0.005; 95% CI, −0.009 to +0.019; DeLong *P* = 0.31; Figure 2A), and the reclassification indices were correspondingly small (continuous NRI, 0.243; 95% CI, −0.187 to 0.673; IDI, 0.009; 95% CI, −0.003 to 0.021). The wide confidence intervals for these indices reflect the limited precision of the incremental estimates. The same pattern was observed on external validation: the baseline-plus-lactate model yielded an AUC of 0.836 (95% CI, 0.791–0.907), compared with 0.821 (95% CI, 0.754–0.888) for the severity baseline, corresponding to a ΔAUC of +0.014 (95% CI, −0.005 to +0.033; DeLong *P* = 0.155; Figure 2B, Table 3). The Brier score for the baseline-plus-lactate model was 0.140 in the development cohort and 0.150 in the external validation cohort (Table 3). Thus, across both cohorts, the prognostic signal carried by lactate was largely already contained within established severity assessment.

**Table 2:** Association between admission lactate and 30-day mortality (multivariable logistic regression).

Indicators	β	SE	OR (95% CI)	*P* value
Non-adjusted model				
Lactate	0.406	0.073	1.50 (1.30–1.73)	<0.001
Adjusted model^*^				
Age (per year)	0.018	0.011	1.02 (1.00–1.04)	0.115
Admission GCS (per point)	−0.223	0.148	0.80 (0.60–1.07)	0.133
Pupillary reactivity (per grade)	0.777	0.291	2.18 (1.23–3.85)	0.008
Rotterdam CT score (per point)	0.914	0.152	2.50 (1.85–3.36)	<0.001
Lactate (per mmol/L)	0.173	0.089	1.19 (1.00–1.42)	0.051

^*^The adjusted model included age, admission GCS, pupillary reactivity, Rotterdam CT score, and admission lactate.

**Figure 2:**
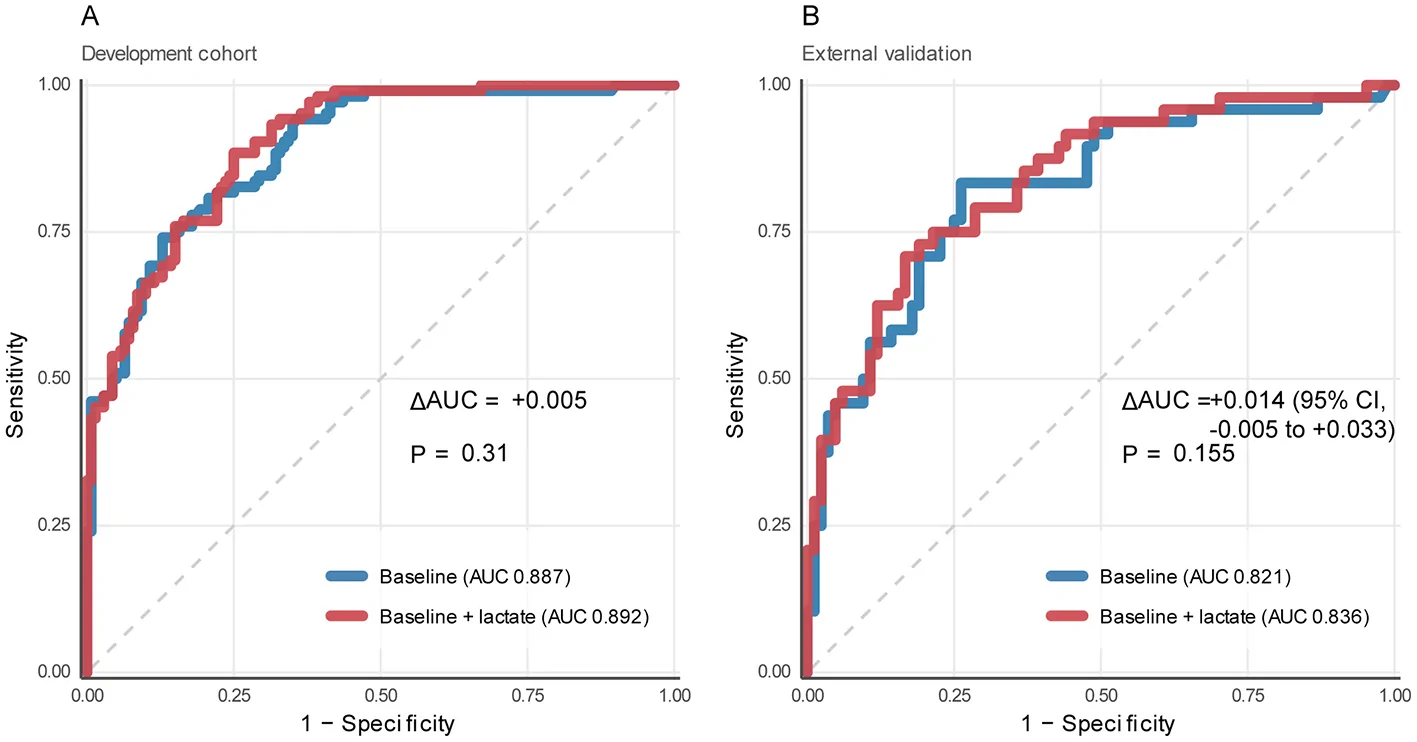
Discrimination and incremental value of admission lactate for 30-day mortality. **(A)** Receiver operating characteristic (ROC) curves in the development cohort comparing three prespecified models: lactate alone (AUC 0.711), the severity baseline of age, Glasgow Coma Scale (GCS), pupillary reactivity, and Rotterdam CT score (AUC 0.887), and the baseline plus lactate (AUC 0.892). Adding lactate to the baseline did not significantly improve discrimination (△AUC = +0.005; DeLong *P* = 0.31). **(B)** ROC curves in the external validation cohort comparing the severity baseline (AUC 0.821) with the baseline plus lactate (AUC 0.836). The incremental change in discrimination was small (△AUC = +0.014; 95% CI, -0.005 to +0.033; DeLong *P* = 0.155).

**Table 3:** Discrimination, overall performance, incremental value, and calibration of the prediction models for 30-day mortality.

Model/metric	Development cohort	External validation cohort
Discrimination, AUC (95% CI)		
Lactate alone	0.711 (0.650–0.768)	0.768 (0.703–0.834)
Baseline model^*^	0.887 (0.845–0.929)	0.821 (0.754–0.888)
Baseline + lactate	0.892 (0.862–0.922)	0.836 (0.791–0.907)
Overall performance		
Brier score, baseline + lactate	0.140	0.150
Internal validation (bootstrap)		
Optimism-corrected C-index (95% CI)	0.884 (0.852–0.916)	—
Calibration slope (95% CI)	0.943 (0.889–0.997)	—
Calibration intercept (95% CI)	0.02 (−0.12 to 0.16)	—
Incremental value of lactate		
ΔAUC (95% CI; DeLong *P*)	+0.005 (−0.009 to +0.019; *P* = 0.31)	+0.014 (−0.005 to +0.033; *P* = 0.155)
Continuous NRI (95% CI)	0.243 (−0.187 to 0.673)	—
IDI (95% CI)	0.009 (−0.003 to 0.021)	—
External calibration (baseline + lactate)		
Calibration slope (95% CI)	—	0.81 (0.69–0.93)
Calibration intercept (95% CI)	—	−0.03 (−0.23 to 0.17)
Observed vs. mean predicted mortality	—	36.8% vs. 35.5%

^*^The baseline model included age, admission GCS, pupillary reactivity, and Rotterdam CT score. The baseline-plus-lactate model additionally included admission lactate. Incremental value was assessed primarily in the development cohort using ΔAUC, continuous NRI, and IDI. NRI and IDI were not calculated in the external validation cohort. AUC, area under the receiver operating characteristic curve; CI, confidence interval; IDI, integrated discrimination improvement; NRI, net reclassification improvement.

### The prognostic model shows good internal but attenuated external calibration

To translate the model into an individualized risk tool, a nomogram incorporating age, GCS, pupillary reactivity, Rotterdam CT score, and lactate was constructed (Figure 3A). On bootstrap internal validation the model showed minimal optimism (optimism-corrected C-index, 0.884; 95% CI, 0.852–0.916) and good internal calibration (calibration slope, 0.943; 95% CI, 0.889–0.997; calibration intercept, 0.02; 95% CI, −0.12 to 0.16; Figure 3B). On external validation, the calibration slope was 0.81 (95% CI, 0.69–0.93), suggesting some degree of overfitting or loss of transportability. The calibration intercept remained close to zero (−0.03; 95% CI, −0.23 to 0.17), and the mean predicted 30-day mortality was similar to the observed mortality (36.8% vs. 35.5%; Figure 3C).

**Figure 3:**
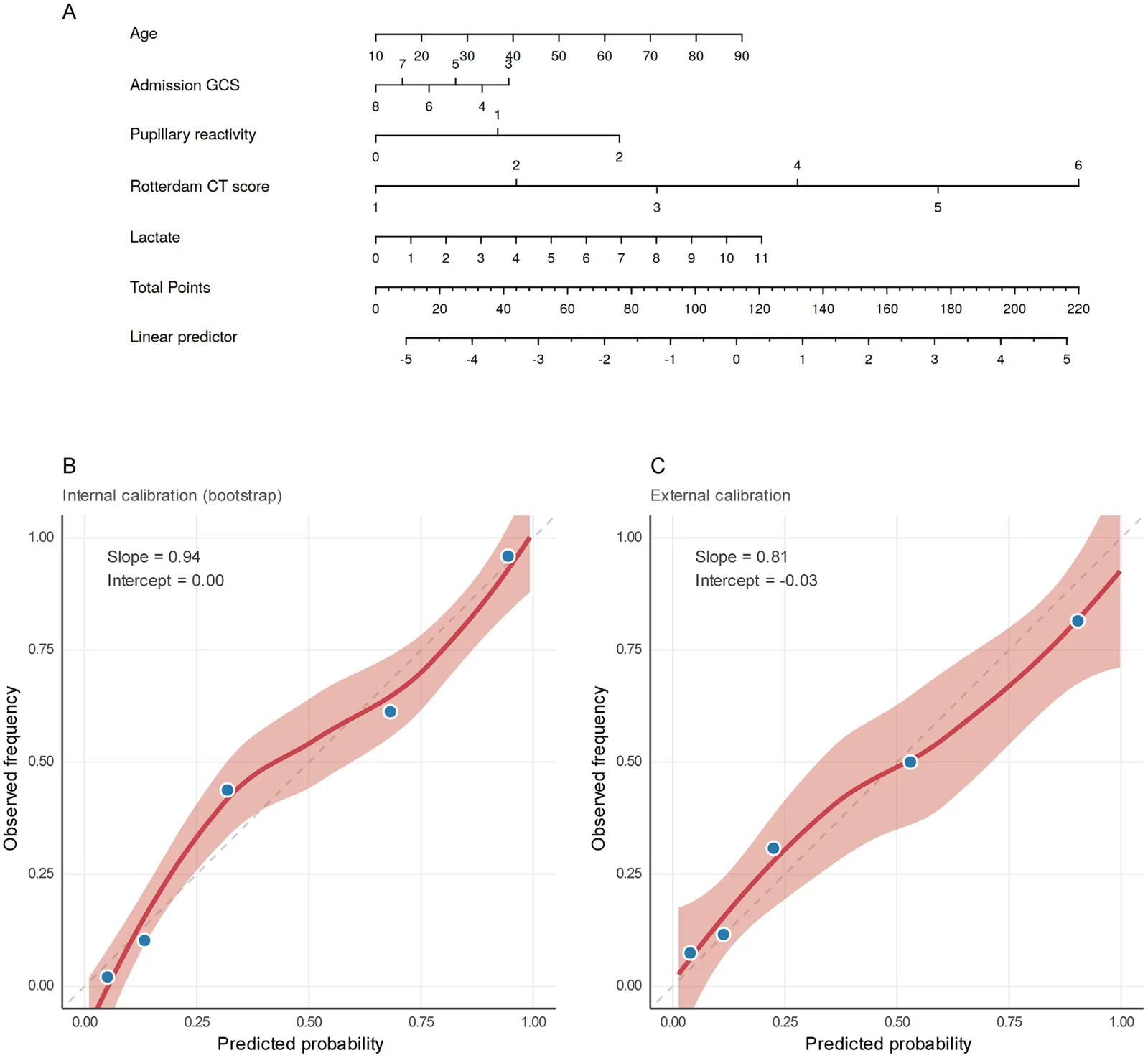
Nomogram and calibration of the prognostic model for 30-day mortality. **(A)** Nomogram for individualized estimation of 30-day mortality risk, integrating age, admission GCS, pupillary reactivity, Rotterdam CT score, and admission lactate. **(B)** Calibration curve of the baseline-plus-lactate model in the development cohort after bootstrap internal validation (optimism-corrected C-index, 0.884; calibration slope, 0.943). **(C)** Calibration curve in the external validation cohort (calibration slope, 0.81; intercept, -0.03). In **(B)** and **(C)**, the dashed diagonal line represents perfect calibration, the solid line indicates the observed calibration, and points denote grouped observations by quintile of predicted risk.

### Lactate shows a graded association but negligible added clinical utility

Decision-curve analysis confirmed the limited incremental value of lactate: across the displayed threshold probability range of 0–0.60, the baseline and baseline-plus-lactate models yielded almost identical net benefit (Figure 4A). Nevertheless, restricted cubic spline analysis showed a graded increase in the adjusted odds of 30-day mortality with increasing lactate, with no significant evidence of nonlinearity (*P* for nonlinearity = 0.396; Figure 4B). Thus, lactate showed a graded association with mortality risk, although it added little to a model already containing established severity markers.

**Figure 4:**
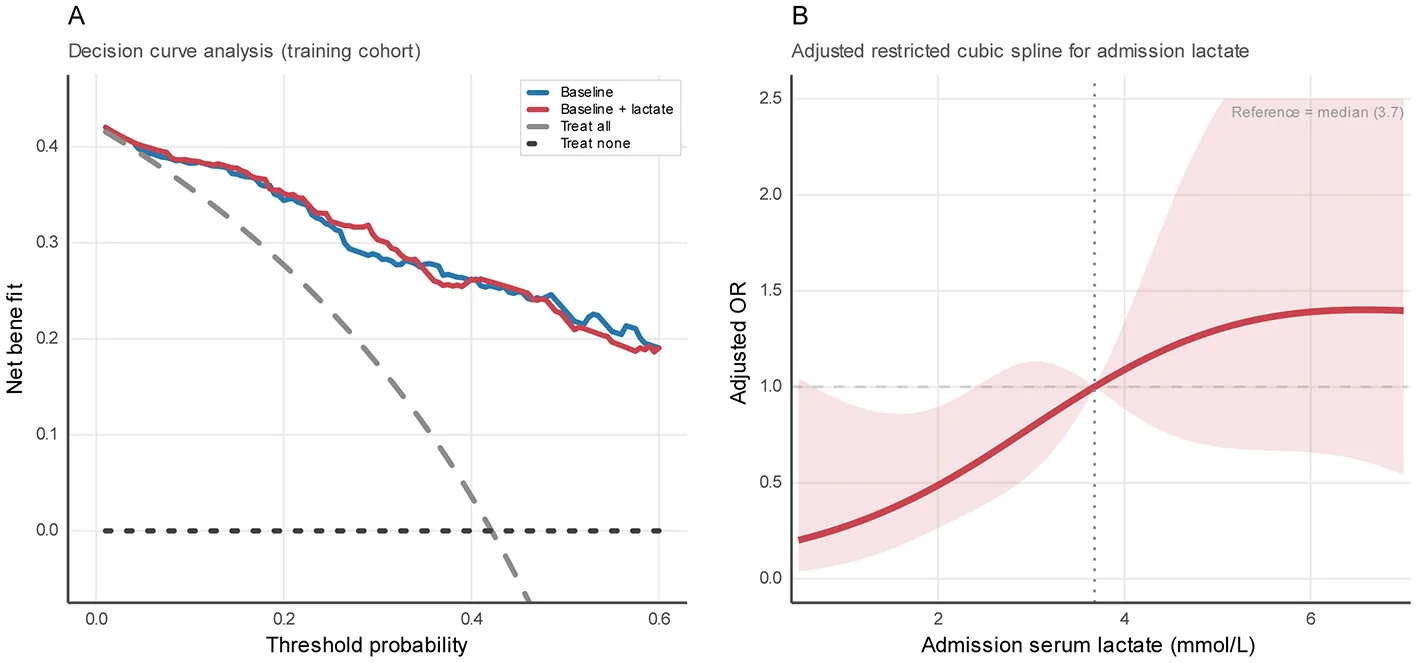
Clinical utility and dose–response relationship of admission lactate. **(A)** Decision curve analysis (DCA) in the development cohort comparing the net benefit of the severity baseline model and the baseline-plus-lactate model across the displayed threshold-probability range of 0−0.60. This range was selected to encompass clinically plausible risk thresholds around and above the observed 30-day mortality rate while avoiding emphasis on the less informative extreme upper-risk range. The “treat-all” (diagonal) and “treat-none” (horizontal) reference lines are also shown. Adding lactate provided negligible additional net benefit. **(B)** Restricted cubic spline (RCS) analysis showing the adjusted association between admission lactate and 30-day mortality. Four knots were placed at the 5th, 35th, 65th, and 95th percentiles of the lactate distribution (0.78, 2.98, 4.40, and 7.49 mmol/L), with the cohort median (3.7 mmol/L) as the reference value. The solid line represents the estimated odds ratio (OR), and the shaded area represents the 95% confidence interval (CI).

### Exploratory effects of lactate on neuronal injury under hypoxic conditions

In exploratory *in vitro* experiments, CCK-8 assays showed a concentration-dependent reduction in viability across 0–20 mmol/L sodium lactate (Figure 5A); 5 mmol/L, approximating the admission lactate of non-survivors, was selected for subsequent experiments. Across the four conditions, lactate alone and hypoxia alone each reduced viability and increased apoptosis relative to control, with the largest effects observed in the combined hypoxia-plus-lactate group (Figures 5B, C). Western blotting showed corresponding changes in Bax, Bcl-2, Caspase-3, LDH-A, and HIF-1α expression, most pronounced in the hypoxia-plus-lactate group (Figure 5D). These exploratory observations suggest that lactate may have biological activity in this *in vitro* system, but they do not establish a causal role in neuronal injury and should be interpreted as hypothesis-generating rather than confirmatory.

**Figure 5:**
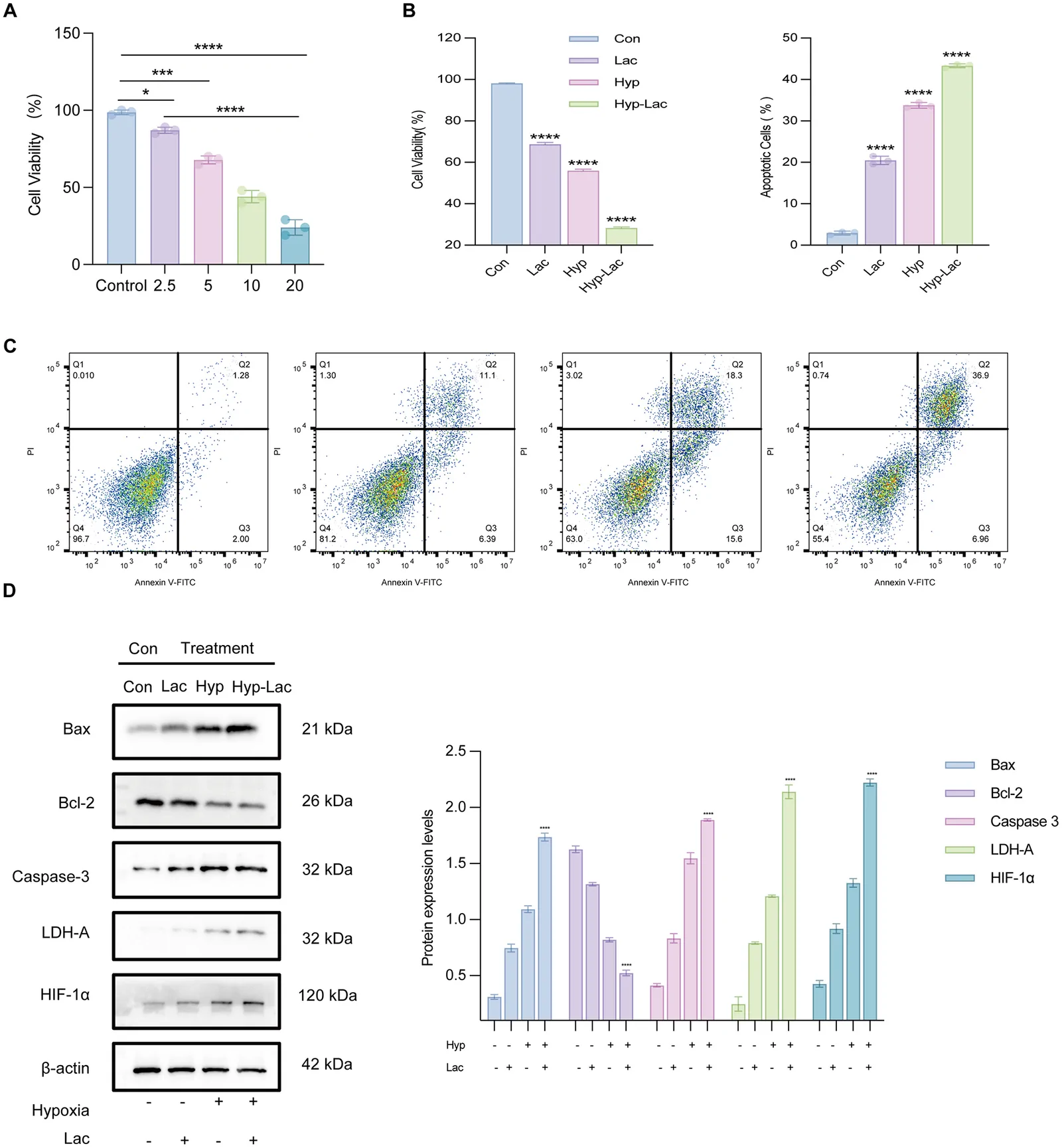
Exploratory effects of lactate on SH-SY5Y cell injury under hypoxic conditions. **(A)** Cell viability of SH-SY5Y cells exposed to graded concentrations of sodium lactate (0, 2.5, 5, 10, and 20 mmol/L), assessed by CCK-8 assay; viability decreased in a concentration-dependent manner, and 5 mmol/L was selected for subsequent experiments. **(B)** Quantitative analysis of cell viability across the four experimental groups: normoxic control (Con), lactate alone (Lac, 5 mmol/L), hypoxia (Hyp), and hypoxia plus lactate (Hyp-Lac). **(C)** Flow cytometric analysis of apoptosis with representative plots and quantification of apoptotic proportions across the four groups. **(D)** Western blot analysis of apoptosis- and metabolism-related proteins (Bax, Bcl-2, Caspase-3, LDH-A, and HIF-1α) with β-actin as the loading control, together with densitometric quantification. Data are presented as mean ± SD from three independent experiments. Concentration-response data were analyzed by one-way ANOVA with Tukey's multiple-comparison test, whereas the four-group experiments were analyzed by two-way ANOVA with hypoxia and lactate as fixed factors followed by Tukey's multiple-comparison test. *P < 0.05, **P < 0.01, ***P < 0.001.

## Discussion

Whether elevated admission lactate serves as an independent prognostic marker or simply reflects the severity of the primary injury in sTBI remains incompletely understood. The present study was designed to address this question and yielded several important findings. First, higher admission lactate was associated with increased 30-day mortality in both the development and external validation cohorts. Second, after adjustment for established indicators of injury severity, including age, pupillary reactivity, GCS score, and Rotterdam CT score, the association between lactate and mortality was substantially attenuated. Third, admission lactate provided only limited incremental prognostic value beyond conventional severity assessment, with negligible improvement in model discrimination. Finally, exploratory *in vitro* experiments showed lactate-associated changes in cell viability, apoptosis, and metabolism-related proteins under hypoxic conditions. These experimental observations are biologically informative but do not establish that circulating lactate causally mediates the clinical association with mortality.

Our findings can be placed within a literature that has reached conflicting conclusions about the prognostic role of admission lactate. Several studies in patients with sTBI or broader TBI populations have reported significant prognostic associations between early lactate levels and mortality, including measurements obtained at hospital admission or during prehospital care (12, 16, 17). A likely source of this discrepancy is how thoroughly injury severity was accounted for: previous studies seldom adjusted simultaneously for pupillary reactivity and the Rotterdam CT score—two of the strongest determinants of outcome after sTBI—and rarely tested their models in an independent cohort. By incorporating both of these severity markers in a single multivariable model and confirming our findings through external validation, we were able to isolate the prognostic contribution of lactate more rigorously than most prior work. Under these conditions, the adjusted OR for lactate was 1.19 per mmol/L (95% CI, 1.00–1.42), indicating a positive association whose confidence interval narrowly includes unity; it added little to model discrimination. We interpret this as suggesting that much of the apparent prognostic signal carried by lactate is shared with established severity markers, rather than as evidence of no independent contribution. That lactate adds limited incremental information once severity is accounted for, however, does not establish that it is biologically inert—leaving open whether it participates actively in the secondary injury process itself.

Notably, a recent prospective study of isolated sTBI reported that admission lactate levels greater than 4 mmol/L independently predicted 30-day unfavorable outcomes (18). Although this finding appears inconsistent with our results, important methodological differences should be considered. That study was restricted to patients with isolated brain injury and adjusted for a different set of covariates. Taken together, these findings suggest that the independent prognostic value of lactate depends largely on the study population and, more importantly, on the severity-related variables included in the final model. When major predictors such as pupillary reactivity and Rotterdam CT score are comprehensively accounted for, as in the present study, the additional prognostic information provided by systemic lactate appears relatively limited.

Cerebral lactate is increasingly recognized as a context-dependent, double-edged metabolite. Through the astrocyte–neuron lactate shuttle, lactate can serve as an alternative energy substrate and signaling molecule for metabolically stressed neurons, and neuroprotective effects have been reported under selected experimental conditions (19, 20). More recently, histone lactylation-mediated upregulation of PSMD14 was shown to alleviate neuronal PANoptosis after traumatic brain injury by activating PINK1-dependent mitophagy (21). Conversely, excessive lactate accumulation during severe metabolic stress may exert detrimental effects. Astrocyte-derived lactate aggravated ischemic brain injury by promoting protein lactylation (22), whereas histone lactylation was recently reported to exacerbate neuronal ferroptosis after traumatic brain injury through a TNFAIP3–ENO1-related mechanism (23). Together, these apparently divergent findings suggest that the effects of lactate depend on the injury context, timing, concentration, oxygen availability, cell type, and downstream lactylation targets.

Our *in vitro* findings fell on the detrimental side of this balance. At concentrations comparable to those observed in non-survivors, lactate alone reduced neuronal viability and altered apoptosis- and metabolism-related markers, with the most pronounced effects observed when combined with hypoxia. These findings suggest that lactate may exert biological effects under specific experimental conditions. However, they should be interpreted independently of the clinical prognostic analysis and considered hypothesis-generating evidence for further mechanistic investigation.

Our results also speak to the broader trajectory of prognostic biomarker research in TBI. The field is moving decisively toward multidimensional characterization: the recent CBI-M framework, for example, integrates clinical, biomarker, imaging, and modifier pillars (24), and brain-specific blood biomarkers such as GFAP and UCH-L1 have been shown in large cohorts (e.g., TRACK-TBI) to add prognostic information beyond clinical models—precisely the independent value that systemic lactate, in our data, lacks (25). Likewise, invasive cerebral microdialysis demonstrates that it is the brain's local metabolic state—the lactate/pyruvate ratio and its temporal dynamics—rather than a single systemic lactate value, that most closely tracks neurological outcome (26). Viewed in this context, our findings argue that the future utility of lactate in sTBI lies not in static, single-timepoint systemic measurement competing against severity scores, but potentially in dynamic or compartment-specific assessment. Consistent with this, time-weighted lactate indices outperform static admission values for ICU mortality prediction (14), and even prehospital lactate measured in the ambulance carries prognostic value for early mortality (17), reinforcing the view that the temporal trajectory of lactate, rather than a single admission value, may carry the information that static measurement misses—and that lactate is best positioned as one inexpensive component of a multimodal panel rather than a stand-alone marker.

Several limitations should be acknowledged. First, the retrospective design introduces the possibility of residual confounding despite multivariable adjustment and multiple imputation. In addition, WLST decisions may have been influenced by the same neurological severity indicators included in the prognostic model, potentially introducing a self-fulfilling prognostic pathway. Although the prespecified sensitivity analysis excluding patients who underwent WLST yielded a directionally consistent association, residual bias related to treatment-limitation decisions cannot be completely excluded. Second, although external validation was performed, both cohorts originated from centers within the same country, and the restrictive eligibility criteria resulted in a selected adult sTBI population. In particular, the exclusion of patients with major extracranial injuries, shock, common metabolic comorbidities, and different treatment trajectories may limit the transportability of our findings to unselected all-comer sTBI populations. Moreover, the exclusion criterion based on craniotomy >5 h after injury may have introduced selection related to post-admission care processes. Third, our exposure measurement was constrained by the retrospective design. Although both centers used the same model of blood gas analyzer (RAPIDLab 1,200), which ensures analytical comparability, we did not have detailed pre-analytical data such as tourniquet time, exact transport duration, or prehospital resuscitation volumes—all of which may influence admission lactate levels independently of TBI severity. These unmeasured pre-analytical factors may have introduced non-differential measurement error that could attenuate the observed lactate–outcome association. This further supports our cautious interpretation that lactate provided limited incremental prognostic value in our selected cohorts and severity-adjusted model, rather than a definitive conclusion about its biological independence. Additionally, only admission lactate levels were analyzed, whereas dynamic measures such as lactate clearance may provide additional prognostic information. Fourth, our adjustment set was limited to neurological severity markers and did not include circulatory or metabolic variables. Finally, the experimental findings were derived from an undifferentiated SH-SY5Y hypoxia model without dedicated sodium-, pH-, or osmolality-matched controls and should therefore be considered exploratory and hypothesis-generating pending validation in more physiologically relevant models.

## Conclusion

In these two selected adult sTBI cohorts, admission lactate was associated with 30-day mortality but provided little incremental prognostic information beyond a neurological severity model comprising age, GCS, pupillary reactivity, and Rotterdam CT score. These findings support interpreting admission lactate as a readily available adjunctive marker rather than a stand-alone prognostic factor. The exploratory *in vitro* findings should be considered hypothesis-generating and do not establish a causal role for circulating lactate in secondary brain injury. Further prospective multicenter studies are warranted to evaluate whether dynamic or multimodal lactate assessment provides additional prognostic value.

## Data Availability

The raw data supporting the conclusions of this article will be made available by the authors, without undue reservation.

## References

[1] Stocchetti N Carbonara M Citerio G Ercole A Skrifvars MB Smielewski P . Severe traumatic brain injury: targeted management in the intensive care unit. Lancet Neurol. (2017) 16:452–64. doi: 10.1016/S1474-4422(17)30118-7 28504109

[2] Jiang JY Gao GY Feng JF Mao Q Chen LG Yang XF . Traumatic brain injury in China. Lancet Neurol. (2019) 18:286–95. doi: 10.1016/S1474-4422(18)30469-1 30784557

[3] Khatri N Sumadhura B Kumar S Kaundal RK Sharma S Datusalia AK. The complexity of secondary cascade consequent to traumatic brain injury: pathobiology and potential treatments. Curr Neuropharmacol. (2021) 19:1984–2011. doi: 10.2174/1570159X19666210215123914 33588734 PMC9185786

[4] Zhang H Gui Y Che W Deng S Yang L. SOCS2 alleviates traumatic brain injury-induced mitochondrial damage and parthanatos in endothelial cells by inhibiting the JAK2/STAT3 signaling pathway. Metab Brain Dis. (2025) 40:289. doi: 10.1007/s11011-025-01724-5 41085788 PMC12521338

[5] Hakiminia B Alikiaii B Khorvash F Mousavi S. Oxidative stress and mitochondrial dysfunction following traumatic brain injury: from mechanistic view to targeted therapeutic opportunities. Fundam Clin Pharmacol. (2022) 36:612–62. doi: 10.1111/fcp.12767 35118714

[6] Hawryluk GWJ Aguilera S Buki A Bulger E Citerio G Cooper DJ . A management algorithm for patients with intracranial pressure monitoring: the seattle international severe traumatic brain injury consensus conference (SIBICC). Intensive Care Med. (2019) 45:1783–94. doi: 10.1007/s00134-019-05805-9 31659383 PMC6863785

[7] Carney N Totten AM O'Reilly C Ullman JS Hawryluk GW Bell MJ . Guidelines for the management of severe traumatic brain injury, fourth edition. Neurosurgery. (2017) 80:6–15. doi: 10.1227/NEU.0000000000001432 27654000

[8] Teasdale G Maas A Lecky F Manley G Stocchetti N Murray G. The glasgow coma scale at 40 years: standing the test of time. Lancet Neurol. (2014) 13:844–54. doi: 10.1016/S1474-4422(14)70120-6 25030516

[9] Bouras M Pageau M Gagnon MA Costerousse O Demers K Grenier-Gagnon A . Association between neurofilament light chain concentrations and outcomes in patients with moderate to severe traumatic brain injury: a systematic review and meta-analysis. Crit Care. (2026). doi: 10.1186/s13054-026-06117-3 42083049 PMC13147746

[10] Deulkar P Singam A Mudiganti VNKS Jain A. Lactate monitoring in intensive care: a comprehensive review of its utility and interpretation. Cureus. (2024) 16:e66356. doi: 10.7759/cureus.66356 39246930 PMC11379417

[11] Belu A Filip N Trandafir LM Spoială EL Ţarcă E Zamosteanu D . Lactate, an essential metabolic marker in the diagnosis and management of pediatric conditions. Diagnostics. (2025) 15:816. doi: 10.3390/diagnostics15070816 40218166 PMC11988452

[12] Jiang H Zeng W Zhang X Dong L Pei Y Peng A. Associations of blood pressure and serum lactate levels upon admission with one-week mortality in patients with severe traumatic brain injury: a multi-center retrospective study. Turk Neurosurg. (2025) 35:492–502. 40420765 10.5137/1019-5149.JTN.47272-24.1

[13] Sharma B Jiang W Dhole Y Agriantonis G Bhatia ND Shafaee Z . Lactate is a strong predictor of poor outcomes in patients with severe traumatic brain injury. Biomedicines. (2024) 12:2778. doi: 10.3390/biomedicines12122778 39767684 PMC11673745

[14] Deininger MM Ralser M Haehn N Huehn M Ziles D Marx G . Time-weighted lactate and glucose-lactate ratio outperform static values in ICU mortality prediction after traumatic brain injury: a retrospective cohort study. J Intensive Care. (2026) 14:26. doi: 10.1186/s40560-026-00864-9 41654972 PMC12977792

[15] Collins GS Reitsma JB Altman DG Moons KG. Transparent reporting of a multivariable prediction model for individual prognosis or diagnosis (TRIPOD): the TRIPOD statement. Bmj. (2015) 350:g7594. doi: 10.1136/bmj.g7594 25569120

[16] Yucel N Ozturk Demir T Derya S Oguzturk H Bicakcioglu M Yetkin F. Potential risk factors for in-hospital mortality in patients with moderate-to-severe blunt multiple trauma who survive initial resuscitation. Emerg Med Int. (2018) 2018:6461072. doi: 10.1155/2018/6461072 30595921 PMC6282151

[17] Martin-Rodriguez F Sanz-Garcia A Lopez-Izquierdo R Delgado Benito JF Martínez Fernández FT Otero de la Torre S . Prehospital lactate levels obtained in the ambulance and prediction of 2-day in-hospital mortality in patients with traumatic brain injury. Neurology. (2024) 103:e209692. doi: 10.1212/WNL.0000000000209692 39088773

[18] Kumar Shukla R Goyal K Mahajan C. Serum lactate, base deficit and blood glucose levels as prognostic markers of isolated severe traumatic brain injury: a prospective observational study. Med J Armed Forces India. (2025). doi: 10.1016/j.mjafi.2025.06.005

[19] Wolahan SM Mao HC Real C Vespa PM Glenn TC. Lactate supplementation in severe traumatic brain injured adults by primed constant infusion of sodium L-lactate. J Neurosci Res. (2018) 96:688–95. doi: 10.1002/jnr.24085 28543565 PMC5696121

[20] Magistretti P J Allaman I. Lactate in the brain: from metabolic end-product to signalling molecule. Nat Rev Neurosci. (2018) 19:235–49. doi: 10.1038/nrn.2018.19 29515192

[21] Xu L Ye Y Gu W Xu X Chen N Zhang L . Histone lactylation stimulated upregulation of PSMD14 alleviates neuron PANoptosis through deubiquitinating PKM2 to activate PINK1-mediated mitophagy after traumatic brain injury. Autophagy. (2025) 21:1473–91. doi: 10.1080/15548627.2025.2471633 40000916 PMC12283019

[22] Xiong XY Pan XR Luo XX Wang YF Zhang XX Yang SH . Astrocyte-derived lactate aggravates brain injury of ischemic stroke in mice by promoting the formation of protein lactylation. Theranostics. (2024) 14:4297–317. doi: 10.7150/thno.96375 39113798 PMC11303085

[23] Zhou L Xu Z Hou X Ren H Ni M Gao L . Histone lactylation exacerbates neuronal ferroptosis after traumatic brain injury: the effect of the upregulation of TNFAIP3 by mediating mitochondrial damage through the deubiquitination of ENO1. Free Radic Biol Med. (2026) 253:561–77. doi: 10.1016/j.freeradbiomed.2026.05.289 42178000

[24] Manley GT Dams-O'Connor K Alosco ML Awwad HO Bazarian JJ Bragge P . A new characterisation of acute traumatic brain injury: the NIH-NINDS TBI classification and nomenclature initiative. Lancet Neurol. (2025) 24:512–23. 40409315 10.1016/S1474-4422(25)00154-1

[25] Korley FK Jain S Sun X Puccio AM Yue JK Gardner RC . Prognostic value of day-of-injury plasma GFAP and UCH-L1 concentrations for predicting functional recovery after traumatic brain injury in patients from the US TRACK-TBI cohort: an observational cohort study. Lancet Neurol. (2022) 21:803–13. doi: 10.1016/S1474-4422(22)00256-3 35963263 PMC9462598

[26] Timofeev I Carpenter KL Nortje J Al-Rawi PG O'Connell MT Czosnyka M . Cerebral extracellular chemistry and outcome following traumatic brain injury: a microdialysis study of 223 patients. Brain. (2011) 134:484–94. doi: 10.1093/brain/awq353 21247930

