# Supplementary material for: Is admission lactate an independent prognostic marker or a reflection of injury severity in severe traumatic brain injury?
Source: Front Neurol. 2026 Oct 19;17:1920098. doi: 10.3389/fneur.2026.1920098 (PMC13724394; doi:10.3389/fneur.2026.1920098)
Supplement: Supplementary file 1 [file Table_1.docx]

**Table S1. Baseline characteristics by 30-day mortality in the development cohort.**

| **Variables** | **Non-survivors (n = 121)** | **Survivors (n = 166)** | **P value** |
| --- | --- | --- | --- |
| Age, years (mean ± SD) | 56.1±14.7 | 49.3±14.7 | <0.001 |
| Male sex, n (%) | 94 (77.7) | 139 (83.7) | 0.254 |
| Weight, kg (mean ± SD) | 66.4±12.4 | 66.5±12.8 | 0.963 |
| Injury-to-NICU time, h, median (IQR) | 3.5 (2.0–4.5) | 3.0 (2.0–4.5) | 0.246 |
| Temperature, °C (mean ± SD) | 36.6±0.6 | 36.8±0.6 | 0.009 |
| Admission GCS, median (IQR) | 6.0 (5.0–6.0) | 7.0 (6.0–8.0) | <0.001 |
| Pupillary reactivity (both/one/none), n | 37/58/17 | 116/27/6 | <0.001 |
| Rotterdam CT score, median (IQR) | 4.0 (3.0–5.0) | 2.0 (2.0–3.0) | <0.001 |
| Emergency surgery, n (%) | 79 (65.3) | 96 (57.8) | 0.247 |
| Admission lactate, mmol/L, median (IQR) | 4.5 (3.4–5.6) | 3.0 (1.8–4.3) | <0.001 |

*Continuous variables: mean ± SD (t test) or median (IQR) (Mann–Whitney U test); categorical: n (%) (χ² test). Pupillary reactivity available in 261 and Rotterdam CT score in 270 of 287 patients owing to missing records; pupillary reactivity: both reactive/one fixed/both fixed. GCS, Glasgow Coma Scale; IQR, interquartile range.*

**Table S2. Baseline characteristics by 30-day mortality in the external validation cohort.**

| **Variables** | **Non-survivors (n = 68)** | **Survivors (n = 117)** | **P value** |
| --- | --- | --- | --- |
| Age, years (mean ± SD) | 58.9±16.4 | 51.1±14.3 | 0.001 |
| Male sex, n (%) | 53 (77.9) | 80 (68.4) | 0.220 |
| Weight, kg (mean ± SD) | 64.8±9.3 | 63.7±12.7 | 0.484 |
| Injury-to-NICU time, h, median (IQR) | 4.5 (3.0–5.6) | 3.0 (2.0–4.5) | <0.001 |
| Temperature, °C (mean ± SD) | 36.6±0.5 | 36.8±0.6 | 0.072 |
| Admission GCS, median (IQR) | 6.0 (5.0–7.0) | 7.0 (6.0–8.0) | <0.001 |
| Pupillary reactivity (both/one/none), n | 27/15/16 | 69/18/8 | 0.001 |
| Rotterdam CT score, median (IQR) | 4.0 (3.0–5.0) | 2.0 (2.0–3.0) | <0.001 |
| Emergency surgery, n (%) | 43 (63.2) | 70 (59.8) | 0.763 |
| Admission lactate, mmol/L, median (IQR) | 4.2 (3.4–5.8) | 2.6 (1.6–3.8) | <0.001 |

*Continuous variables: mean ± SD (t test) or median (IQR) (Mann–Whitney U test); categorical: n (%) (χ² test). Pupillary reactivity available in 153 and Rotterdam CT score in 161 of 185 patients owing to missing records; pupillary reactivity: both reactive/one fixed/both fixed. GCS, Glasgow Coma Scale; IQR, interquartile range.*

**Table S3. Sensitivity analysis: association between admission lactate and 30-day mortality after excluding patients with withdrawal of life-sustaining treatment (WLST).**

| **Analysis** | **n (deaths)** | **Adjusted lactate OR (95% CI) *** | **P value** |
| --- | --- | --- | --- |
| Main analysis (all patients) | 287 (121) | 1.19 (1.00–1.42) | 0.051 |
| Excluding WLST patients | 247 (91) | 1.23 (1.01–1.50) | 0.036 |

** Adjusted for age, admission GCS, pupillary reactivity, and Rotterdam CT score (multiply-imputed). Results were directionally consistent after exclusion of patients who underwent WLST.*

**Table S4.** **Missing data for each variable in the development and external validation cohorts, stratified by 30-day outcome.**

| **Variable** | **Development cohort** | | **External validation cohort** | |
| --- | --- | --- | --- | --- |
|  | **Survivors (n = 166)** | **Non-survivors (n = 121)** | **Survivors (n = 117)** | **Non-survivors (n = 68)** |
| Age | 0 (0%) | 0 (0%) | 0 (0%) | 0 (0%) |
| Sex | 0 (0%) | 0 (0%) | 0 (0%) | 0 (0%) |
| Body weight | 0 (0%) | 0 (0%) | 0 (0%) | 0 (0%) |
| Admission GCS | 0 (0%) | 0 (0%) | 0 (0%) | 0 (0%) |
| Pupillary reactivity | 17 (10.2%) | 9 (7.4%) | 22 (18.8%) | 10 (14.7%) |
| Rotterdam CT score | 9 (5.4%) | 8 (6.6%) | 14 (12.0%) | 10 (14.7%) |
| Admission lactate | 0 (0%) | 0 (0%) | 0 (0%) | 0 (0%) |
| Body temperature | 5 (3.0%) | 4 (3.3%) | 10 (8.5%) | 5 (7.4%) |
| Injury-to-admission time | 0 (0%) | 0 (0%) | 0 (0%) | 0 (0%) |
| Emergency surgery | 0 (0%) | 0 (0%) | 0 (0%) | 0 (0%) |

*Values are number of missing observations (% within each 30-day outcome group). GCS, Glasgow Coma Scale.*

**Table S5. Comparison of study centers and cohort characteristics.**

| **Characteristic** | **Development cohort (Yangzhou)** | **External validation cohort (Qinghai)** |
| --- | --- | --- |
| Location | Yangzhou, Jiangsu (eastern China) | Xining, Qinghai (western China) |
| Elevation | ~5 m | ~2,260 m |
| Catchment population | Predominantly urban | Mixed urban-rural |
| Prehospital transport time | Generally <60 min | Often >120 min |
| ICU setting | General ICU with neurosurgical consultation | Dedicated NICU |
| ICP monitoring | Available per clinical indication | Standardized protocol |
| Study period | January 2018 – December 2022 | January 2019 – December 2023 |
| Sample size | 287 | 185 |
| 30-day mortality | 121 (42.2%) | 68 (36.8%) |

**Table S6. Characteristics of withdrawal of life-sustaining treatment stratified by center and 30-day outcome.**

| **Variables** | **Development cohort (n = 287)** | | **External validation cohort (n = 185)** | |
| --- | --- | --- | --- | --- |
|  | **Survivors** **(n = 166)** | **Non-survivors** **(n = 121)** | **Survivors** **(n = 117)** | **Non-survivors** **(n = 68)** |
| **WLST, n (%)** | 10 (6.0) | 30 (24.8) | 3 (2.6) | 24 (35.3) |
| Time from admission to WLST, days, median (IQR) | 5.0 (4.3–5.8) | 3.0 (2.0–4.0) | 4.0 (4.0–5.0) | 4.0 (3.0–5.0) |
| **Reason for WLST, n (% of WLST patients)** |  |  |  |  |
| Family wishes | 5 (50.0) | 21 (70.0) | 2 (66.7) | 10 (41.7) |
| Poor prognosis | 5 (50.0) | 4 (13.3) | 1 (33.3) | 13 (54.2) |
| Other | 0 (0) | 5 (16.7) | 0 (0) | 1 (4.2) |

*WLST, withdrawal of life-sustaining treatment; IQR, interquartile range.*
